# Some Considerations of the Dental Outlook in Cuba

**Published:** 1901-05

**Authors:** Erastus Wilson


### SOME CONSIDERATIONS OF THE DENTAL OUTLOOK IN CUBA.[1]

BY ERASTUS WILSON, M.D.[2]

It is hoped and expected that the Aurora of the twentieth century will awaken many innovations in the island of Cuba. Among others, we, for the first time, are making efforts towards organizing the profession of dentistry upon a scientific basis.

Hitherto, although a limited number of dentists who have had the advantage of a scientific training in the United States (mostly Cubans) have been practising here, the vast majority have been working at it as a handicraft or trade, which, from their point of view, requires little time for preparation in order to acquire the slight knowledge and skill of their teachers who had obtained their own preparation in the same way.

Indeed, several of these teachers had no, or very few, dental patients, but gained their livelihood by the fees paid them by their pupils, and by the transient public attracted to them by their skill in advertising and in obtaining authority from former venal governments to dispense diplomas as part of their business. The island is now pretty well stocked with diplomas of their origin, whose possessors offer the public cheap prices in correspondence with the quality of their services.

These chaotic conditions are to be principally charged to account of the deranged and disorganized state of our society, due to two destructive wars and the complete breaking up of our former social system, the final destruction of the antique moulds in which society was cast, and the sudden annihilation of our former wealth, which had been developed by forced labor of human chattels.

These disastrous cataclysms disjointed everything here, and in the resulting chaos many of the unemployed caught at every floating straw that seemed to offer them a means of living, and many, no doubt, believed that by a short tuition in the aforesaid extemporized schools of dentistry they could acquire a profession that would bring them wealth, while in order to learn any other

---

[1] Read before the Third Pan-American Medical Congress, held in Havana, February 4, 5, 6, and 7, 1901.

[2] President of Section of Oral and Buccal Surgery, P.-A.D.C.

trade, several years would be necessary to attain to the acquired skill. Thus from all this class of recruits to our ranks, our calling takes a lower grade than any other handicraft or trade.

Under these circumstances it need not surprise any one if many of these recruits are illiterates of low class, or that the majority of those of our calling in this island are not capable of exemplifying the best service our modern profession is able to give to the public.

As it is always the majority of any profession or art that determines its social category, it must be the persistent effort of the better class of our members to multiply their proportional numbers by a higher class of recruits, which fortunately our new scheme of public instruction somewhat favors.

In it dentistry finds a place in the official curriculum of the University of Habana, although it is there represented by only two professorships, and the selection of these two professors has been considerably controlled by antique habits of thought formed under the old influences and by an insane Chauvinism or race jealousy here still extant.

Although there are here several Cuban dentists well qualified for professorships, with diplomas from some of the leading American dental colleges, who were available and desirous of occupying them, none of these were given the most important of the two professorships.

We may, however, consider it one step in the right direction to obtain a place for our art in the university classes. It is now presumable that it will guard the door to our profession, which has until now been wide open to all comers, however illiterate, who could produce a few five dollar pieces of money, as key to the situation. Some literary qualifications will henceforth be required, and a less number of abuses will creep in; but the dental class as now organized in our University will not be able to graduate competent alumni; compelling them, as it does, to acquire in the regular medical classes the fundamental elements of medicine will not alone fit them as dentists. If it were so, then every regularly educated physician would be a dentist of competent skill. A few hours each day in the interval between the medical classes dedicated to the dental operating-room clinics and mechanical laboratory are not sufficient preparation for dental practice.

Our specialty is eminently a branch of preventive medicine both in its operative and prosthetic aspects; but, although our students should be well grounded in the elements of general medicine and the special buccal pathology and therapeutics, far the major part of dental service to society is dedicated to the conservation of the natural organs and the construction of efficient and artificial substitutes for such of the dental organs as may have been lost by want of proper attention to their care. A proper education in these practical parts of our profession can only be acquired by several years of constant clinical exercises under skilful instructors.

Dental education has been organized in the Habana University by authorities not connected with our specialty, and so far as I know without consultation with any of its members; but I regard it as one step towards a proper organization of our profession here, and for which we ought to be duly grateful and are in duty bound to give it moral support, but with persistent recommendations for a better organization.

My own idea is that at least two years of previous technical training in the laboratory of some well-known dentist and a certificate of proficiency should be one of the requirements for admission to University classes.

Thus the twentieth century opens with encouraging prospects for our island in all its aspects, and imposes upon us serious obligations to the fundamental laws of human progress.

It is our solemn duty to organize associative scientific efforts to generalize in the public mind a knowledge and appreciation of higher standards of excellence in dental operations and honorable professional dealings in order that our art may attain the legitimate social position to which its merits entitle it.

We are now face to face with this prime necessity, and in our first steps in its direction we meet with a grave obstacle that must be contended with. It is that horde of intruders, with no sufficient instruction nor regular training, many of which are in possession of the certificates and diplomas above referred to, and to which I attribute scant scientific significance, but on the authority of which they claim to be members of our profession.

We have here a certain and increasing number of competent dentists who fear to organize themselves together as such, in contradistinction to those, lest they appeal to the excitable Chauvinism or patrioteria which is now rampant in our island.

In the social organization of our profession here must we accredit with the public these irregulars by recognizing them as fellows, while all their operations and their current public advertisements are discreditable to us as a profession? Would such a recognition advance our professional credit? or would it not rather be a public acceptance by us of the low standard of excellence propagated by these parties? Science knows no political limits; but here political and racial jealousy occupies a menacing attitude unless we assume a conciliatory tolerance of the vestiges of the ancient *régime.*

What appears to me a practicable solution of this difficulty is the following,—viz.:

All those at present among us who have had a scientific training in our profession have obtained it in American colleges, consequently must be well grounded in the English language, and although dentistry has now a place in our University, several years must pass before it can graduate its alumni,—this without consideration of its infantile and imperfect organization; therefore we may organize in two separate dental societies, one English speaking and the other Spanish speaking, so that one may in no way antagonize or interfere with the proceedings of the other. In this way would be two distinct categories which would operate as a continuous stimulant to those of one category to attain a competent degree of culture to enable it to secure recognition and entrance into the other, thus tending towards a continuous improvement in our merits and social standing in this community.

We have now a sufficient number of American graduates here to assure the rapid generalization of the greater proportion of the better class of dental work, which would soon raise the credit of our profession in the general estimation and will gradually force the cheap Johns into the background.

This Pan-American Congress will surely mark the inauguration of a new era here, social, professional, industrial, and agricultural.

Let us do our part of the social obligation to this new era, associating ourselves in organized form in order to facilitate and strengthen our efforts in fulfilment of this social duty. Then we may accredit ourselves with our inner consciousness and with outer society, and rise in the social scale while accrediting the better class of dental service at reasonable fees, which, however, must ever be more or less graduated to correspond with the financial ability of each patient, as this is both reasonable and honorable.

Raise the quality of dental work, and prices will raise themselves to the highest limits of appreciation and financial ability of each client.

